# Subclavian steal syndrome: a case study of diagnosis, management, and successful surgical resolution

**DOI:** 10.1093/jscr/rjae280

**Published:** 2024-05-03

**Authors:** Marah Mansour, Lutfallah Raffoul, Omar Alattar, Hala Deeb, Laila Albainy, Saleh Taqem

**Affiliations:** Faculty of Medicine, Tartous University, 8th March street, 7th project, Tartous, Syrian Arab Republic; Division of Colon and Rectal Surgery, Department of Surgery, Mayo Clinic, 200 First St. SW Rochester, MN 55905, United States; Faculty of Medicine, Al Andalus University for Medical Sciences, Al-Qadmus street, Tartous, Syrian Arab Republic; Faculty of Medicine, Damascus University, Fayez Mansour street, Damascus, Syria; Faculty of Medicine, Damascus University, Fayez Mansour street, Damascus, Syria; Department of Plastic Surgery, Al Mujtahid hospital, Khaled Ibn Alwalid street, Damascus, Syria; Department of Cardiology, Alasad University hospital, 17 April street, Damascus, Syria

**Keywords:** subclavian steal syndrome, SSS, carotid-vertebral bypass, TIA, case report

## Abstract

Subclavian steal syndrome (SSS) is a rare vascular condition characterized by retrograde blood flow in the vertebral artery, often discovered incidentally in asymptomatic patients. We present a 65-year-old male with recurrent transient ischemic attacks (TIAs) attributed to 99% stenosis at the origin of the left subclavian artery, leading to SSS. Diagnostic modalities included duplex ultrasound, confirming inverted left vertebral artery flow, and multi-slice computed tomography angiography, confirming the diagnosis. Despite an unsuccessful attempt at balloon angioplasty, successful vascular surgery was performed, establishing a left carotid-vertebral artery bypass. The patient recovered well without complications. This case underscores the importance of considering SSS in TIA cases, utilizing non-invasive diagnostic tools, and highlighting the successful management of symptomatic SSS through surgical intervention.

## Introduction

Subclavian steal syndrome (SSS) is a vascular condition characterized by retrograde flow in the vertebral artery (VA) away from the brain stem, which results in vertebrobasilar insufficiency [1–8]. It can manifest with various neurological and vascular symptoms, such as paroxysmal vertigo, drop episodes, and/or arm claudication. This phenomenon is discovered accidentally in asymptomatic patients [1, 5]. SSS commonly arises from high-grade stenosis or blockage of the subclavian artery (SCA), causing a SCA pressure differential that reverses blood flow in the ipsilateral vertebral artery (IVA), thereby ‘stealing’ blood from the contralateral subclavian artery. Symptoms can be triggered by vigorous arm movement and sudden head tilting towards the affected side [1, 5–7]. SSS affects 0.6% to 6.4% of the general population, with a higher prevalence in elderly males due to increased atherosclerosis [2, 5]. A 4:1 ratio of left-to-right-side SSS has been observed [5]. Diagnosis involves non-invasive methods such as color-coded Doppler or transcranial Doppler ultrasonography (TDU), and confirmation through ultrasound, computed tomography (CT) or magnetic resonance angiography (MRA) [2, 5–7]. Management may include medical treatment alone or surgical/endovascular revascularization, with percutaneous transluminal angioplasty recommended for symptomatic cases. Surgical procedures like subclavian-carotid transposition, carotid-subclavian bypass procedures, or carotid-axillary bypass are being considered in cases of failed endovascular approaches [2].

## Case presentation

**Figure 1:**
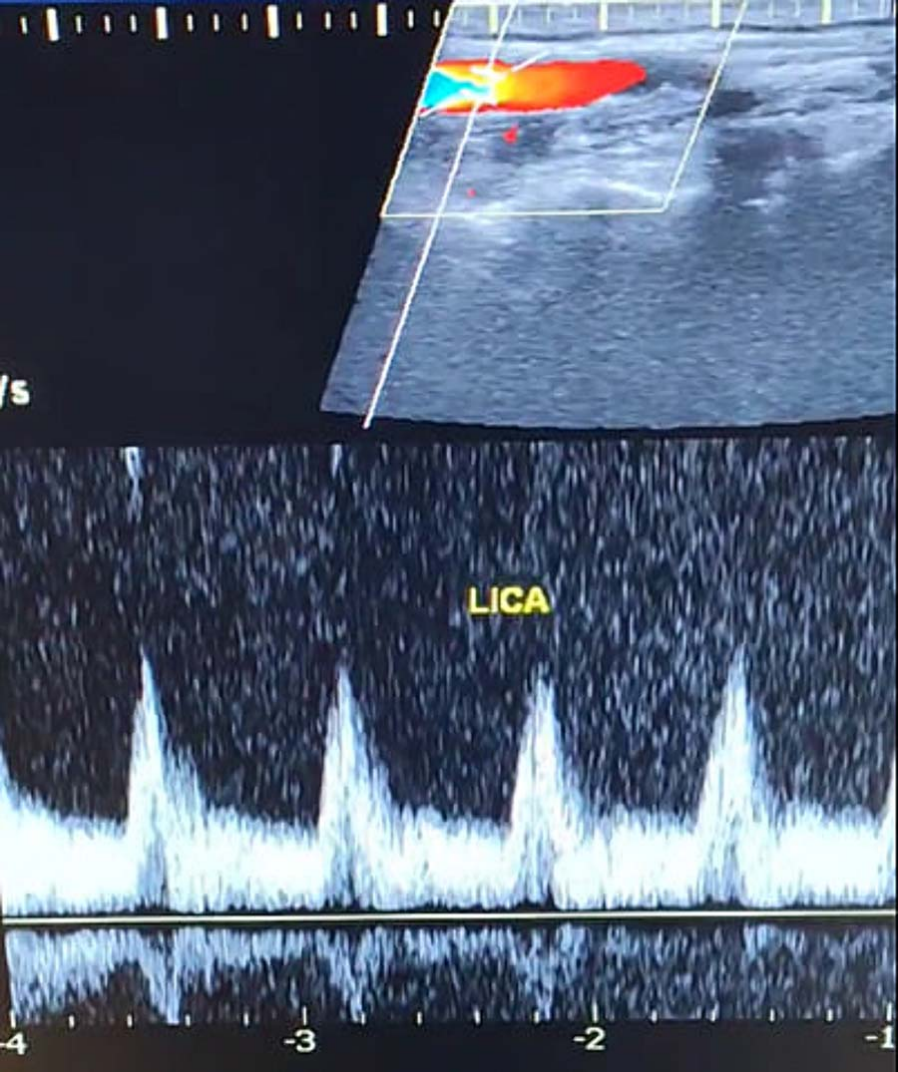
Duplex ultrasound showing a normal flow direction and insignifcant atherosclerotic plaques in the left internal carotid artery.

**Figure 2:**
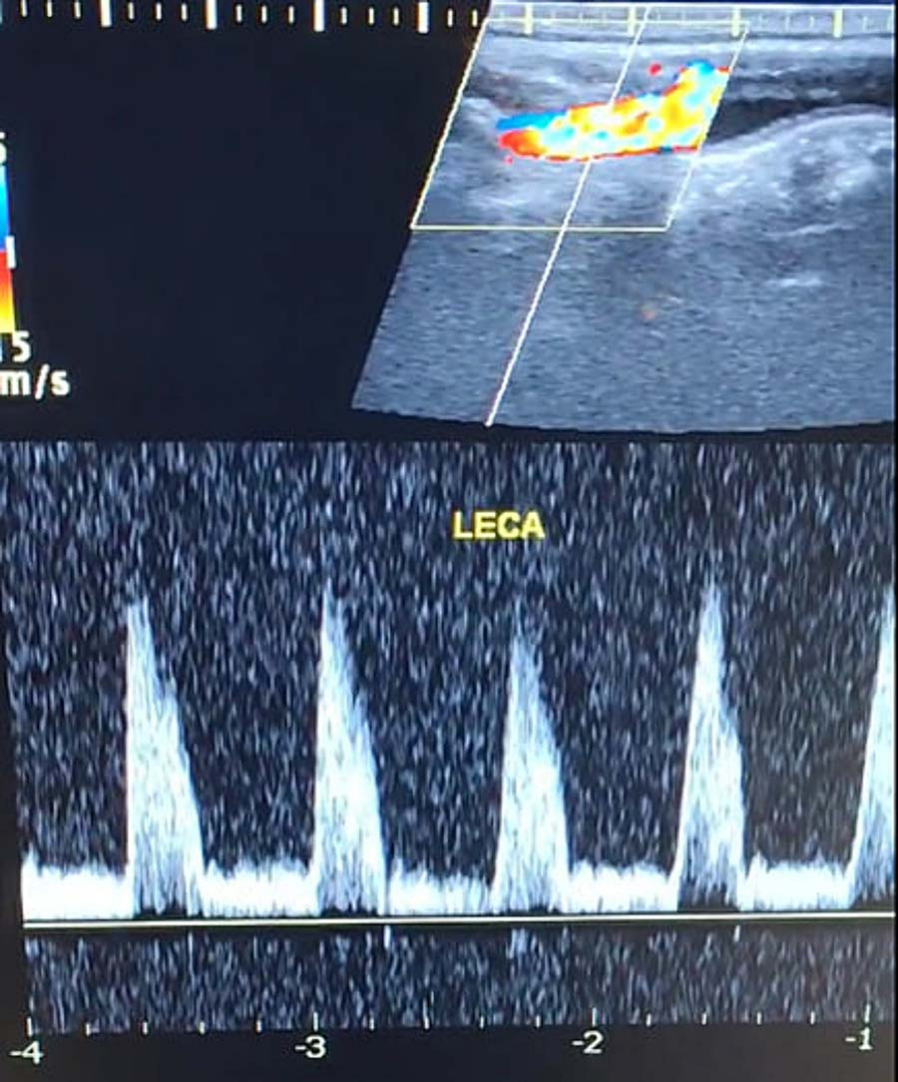
Duplex ultrasound showing a normal flow direction and insignifcant atherosclerotic plaques in the left external carotid artery.

**Figure 3:**
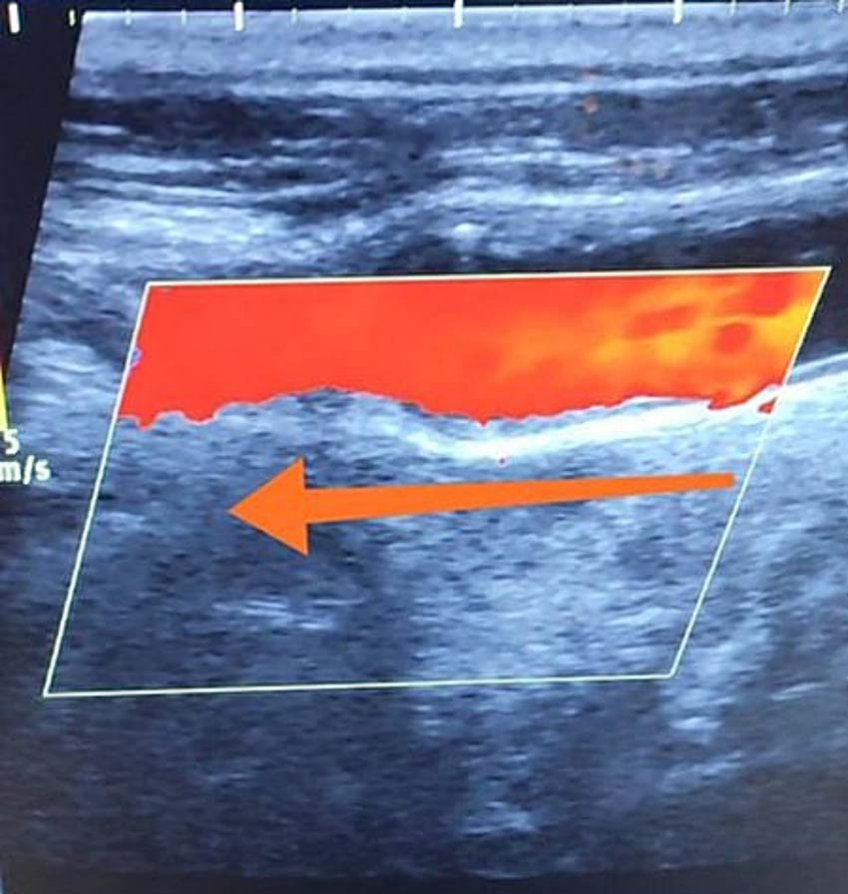
Duplex ultrasound showing a normal flow direction in the common carotid artery.

**Figure 4:**
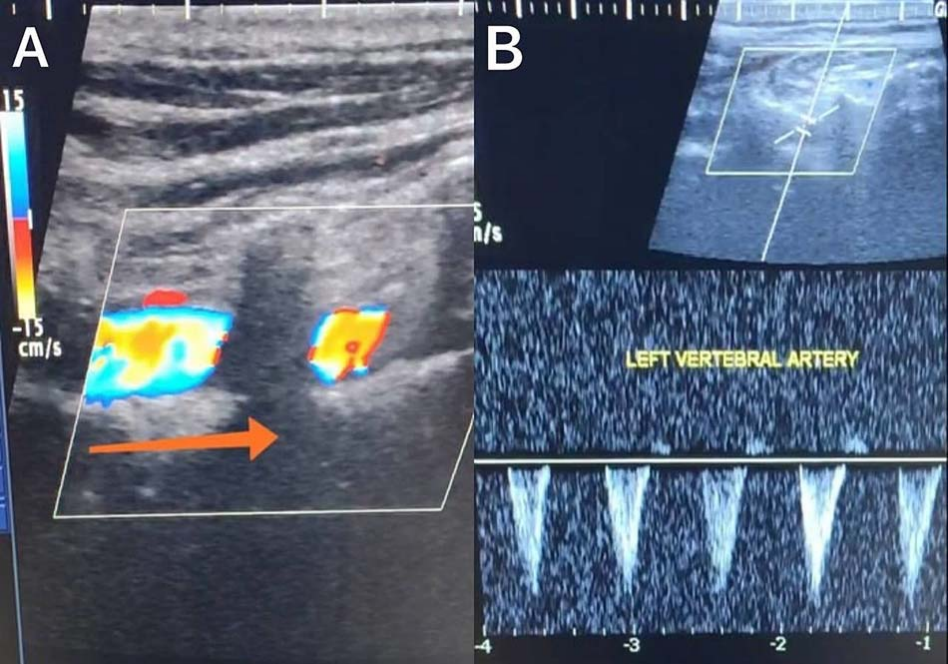
Duplex ultrasound showing a completely inverted flow direction in the left vertebral artery.

**Figure 5:**
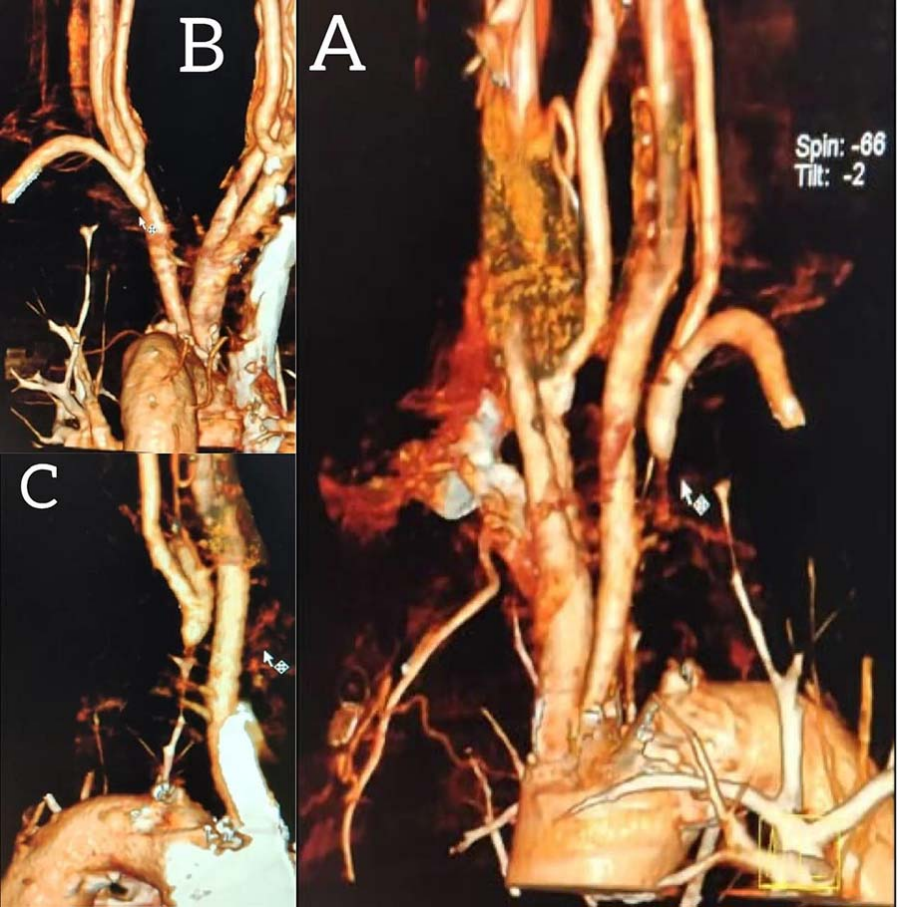
Multi-slice computed tomography angiography demonstrating 99% stenosis at the origin of the left subclavian artery.

A 65-year-old male presented with recurrent transient ischemic attacks (TIAs), characterized by severe balance disturbances, transient alterations in consciousness, and visual obscurations. Clinical examination revealed high blood pressure in the right arm and absent left radial and brachial artery pulses. Vital signs were normal. Laboratory tests are analyzed as follows: urea: 22 mg/dl, glucose: 150 mg/dl, cholesterol: 199 mg/dl, and triglycerides: 222 mg/dl. A medical history of type 2 diabetes mellitus (DT2), hypertension (HTN), hyperlipidemia, ischemic heart disease, and a heavy smoking history were recorded. Five years before presentation, the patient underwent coronary stenting. Medication history included metformin 1000 mg, aspirin 81 mg, rosuvastatin 40 mg, and ramipril 5 mg. Neck duplex ultrasound (DU) showed normal flow direction and insignificant atherosclerotic plaques in the left internal carotid artery (CA) and left external CA (Figs 1 and 2), normal common CA (Fig. 3), a completely inverted flow direction in the left VA (Fig. 4) that suspected a significant ostial lesion in the left SCA. Multi-slice computed tomography angiography demonstrated 99% stenosis at the left SCA origin and confirmed the presence of SSS (Fig. 5). A balloon angioplasty was unsuccessfully attempted to expand the left SCA. A left carotid VA bypass surgery was performed, and a vascular graft was used to establish a blood flow pathway between the left VA and the left CA. By follow-up, the patient was stable without complications.

## Discussion

With a prevalence ranging from 0.6% to 6.4%, SSS is considered a rare phenomenon, and high suspicion is required for diagnosis [2, 5]. The basilar artery is formed by the two vertebral arteries and connects to the brain’s anterior circulation, comprising the Willis circle. However, a steno-occlusive lesion of the proximal SCA may lead to limited blood flow to the upper extremity. Consequently, blood flows through the contralateral VA to the basilar artery, around the circle of Willis, and then descends through the IVA to supply the affected upper extremity, leading to upper limb and vertebrobasilar insufficiency. This altered physiology manifests in signs and symptoms constellation defining SSS [1, 4]. A total of 82.3% of lesions primarily affect the left side as a result of the sharper curvature at the origin of the left SCA, which increases blood flow turbulence and promotes atherosclerotic plaque formation [1, 2, 5–7]. In this case, a patient with retrograde left VA flow on DU is presented (Fig. 4). Most patients are asymptomatic due to developing collateral circulation [2]. However, it may manifest with arterial insufficiency affecting the upper limb (paresthesia, weakness, and claudication), the brain (vertigo, dizziness, diplopia, ataxia, dysarthria, slurred speech, and syncopal episodes) [1, 2, 5, 7], or the heart if the patient has a coronary artery bypass graft supplied by the internal mammary artery [6]. Our patient suffered from recurrent TIA, manifested as severe balance disturbances, transient altered mental status, and amaurosis fugax (transient vision loss). The most common diagnostic tests for SSS are the DU and TDU due to their accessibility and low cost, which show the subsequent retrograde blood flow in the IVA. Additionally, MRA or CT angiography is used as a confirmatory modality [1, 2, 5–8], but the gold-standard is conventional cerebral angiography [1]. A difference in blood pressure readings between arms >20 mmHg is a significant and noticeable sign. In our case, neck DU showed an inverted left VA flow direction (opposite to the carotid) (Fig. 1). MSCT confirmed the diagnosis of SSS, demonstrating a significant ostial lesion in the left SCA. Conservative management is preferred with minimally symptomatic patients by reducing risk factors like a specific regime and treating comorbidities such as: HTN, DT2, hyperlipidemia, and smoking cessation as a part of minimizing morbidity and preventing complications [1, 2, 5–8]. Surgery is recommended for severe and untreated cases through percutaneous intervention or surgical revascularization by using vascular bypass accompanied by angioplasty and stenting [1, 2, 5–7]. In our case, expanding the left subclavian by catheterization did not succeed; however, a left carotid-vertebral bypass was successfully performed. The most common complications are stroke (ranging from 0.4% to 4.7%) due to distal emboli, thrombosis or bleeding and rupture because of aggressive catheter or wire manipulation [1, 7]. In our case, the patient continues on HTN and DT2 medications: ramipril 5 mg, rosuvastatin 40 mg, aspirin 81 mg, and metformin 1000 mg.

## Conclusion

SSS is a rare, asymptomatic phenomenon. This case highlights the successful surgical management of symptomatic SSS.

## Data Availability

Not applicable. All data (of the patient) generated during this study are included in this published article and its supplementary information files.
